# The Association Between Physical Distancing Behaviors to Avoid COVID-19 and Health-Related Quality of Life in Immunocompromised and Nonimmunocompromised Individuals: Patient-Informed Protocol for the Observational, Cross-Sectional EAGLE Study

**DOI:** 10.2196/52643

**Published:** 2024-08-13

**Authors:** Paul Williams, Timothy A Herring, Renata T C Yokota, Tiago Maia, Sudhir Venkatesan, James C Marcus, Gabriella Settergren, Sofie Arnetorp, Andrew Lloyd, Johan L Severens, James W Varni, Sharon Dixon, Lweendo Hamusankwa, Philip A Powell, Sylvia Taylor, John E Ware Jr, Marieke Krol

**Affiliations:** 1 Global Evidence BioPharmaceuticals Medical AstraZeneca Gothenburg Sweden; 2 Patient Centered Solutions IQVIA Courbevoie France; 3 Medical Evidence, Epidemiology, Vaccine & Immune Therapies BioPharmaceuticals Medical AstraZeneca Wilmington, DE United States; 4 P95 Epidemiology & Pharmacovigilance Leuven Belgium; 5 Patient Centered Solutions IQVIA Porto Salvo Portugal; 6 BPM Evidence Statistics, BioPharmaceuticals Medical AstraZeneca Cambridge United Kingdom; 7 Patient Centered Solutions IQVIA Washington, DC United States; 8 Global Evidence Portfolio Delivery BioPharmaceuticals Medical AstraZeneca Gothenburg Sweden; 9 Health Economic & Payer Evidence BioPharmaceuticals Medical AstraZeneca Gothenburg Sweden; 10 Acaster Lloyd Consulting Ltd London United Kingdom; 11 Severens HTA Consultancy Venray Netherlands; 12 Texas A&M University College Station, TX United States; 13 Patient representative Sulphur Springs, TX United States; 14 Patient representative London United Kingdom; 15 Sheffield Centre for Health and Related Research (SCHARR) University of Sheffield Sheffield United Kingdom; 16 Philip A Powell Consulting Sheffield United Kingdom; 17 Medical Evidence, Vaccine & Immune Therapies BioPharmaceuticals Medical AstraZeneca Cambridge United Kingdom; 18 John Ware Research Group Watertown, ME United States; 19 Patient Centered Solutions IQVIA Amsterdam Netherlands

**Keywords:** SARS-CoV-2, social isolation, patient participation, patient-reported outcome measures, quality of life, immunosuppression, respiratory tract infection, cost of illness, surveys and questionnaires, protocol

## Abstract

**Background:**

Immunocompromised individuals are known to respond inadequately to SARS-CoV-2 vaccines, placing them at high risk of severe or fatal COVID-19. Thus, immunocompromised individuals and their caregivers may still practice varying degrees of social or physical distancing to avoid COVID-19. However, the association between physical distancing to avoid COVID-19 and quality of life has not been comprehensively evaluated in any study.

**Objective:**

We aim to measure physical distancing behaviors among immunocompromised individuals and the association between those behaviors and person-centric outcomes, including health-related quality of life (HRQoL) measures, health state utilities, anxiety and depression, and work and school productivity impairment.

**Methods:**

A patient-informed protocol was developed to conduct the EAGLE Study, a large cross-sectional, observational study, and this paper describes that protocol. EAGLE is designed to measure distancing behaviors and outcomes in immunocompromised individuals, including children (aged ≥6 mo) and their caregivers, and nonimmunocompromised adults in the United States and United Kingdom who report no receipt of passive immunization against COVID-19. We previously developed a novel self- and observer-reported instrument, the Physical Distancing Scale for COVID-19 Avoidance (PDS-C19), to measure physical distancing behavior levels cross-sectionally and retrospectively. Using an interim or a randomly selected subset of the study population, the PDS-C19 psychometric properties will be assessed, including structural validity, internal consistency, known-group validity, and convergent validity. Associations (correlations) will be assessed between the PDS-C19 and validated HRQoL-related measures and utilities. Structural equation modeling and regression will be used to assess these associations, adjusting for potential confounders. Participant recruitment and data collection took place from December 2022 to June 2023 using direct-to-patient channels, including panels, clinician referral, patient advocacy groups, and social media, with immunocompromising diagnosis confirmation collected and assessed for a randomly selected 25% of immunocompromised participants. The planned total sample size is 3718 participants and participant-caregiver pairs. Results will be reported by immunocompromised status, immunocompromising condition category, country, age group, and other subgroups.

**Results:**

All data analyses and reporting were planned to be completed by December 2023. Results are planned to be submitted for publication in peer-reviewed journals in 2024-2025.

**Conclusions:**

This study will quantify immunocompromised individuals’ physical distancing behaviors to avoid COVID-19 and their association with HRQoL as well as health state utilities.

**International Registered Report Identifier (IRRID):**

RR1-10.2196/52643

## Introduction

### Background

Recent estimates suggest that there are >14 million immunocompromised individuals in the European Union [1] and approximately 19 million immunocompromised individuals in the United States [2,3]. Relative to their immunocompetent peers, immunocompromised individuals are at an increased risk of SARS-CoV-2 infection [4]. Moreover, despite the availability and use of COVID-19 vaccines, immunocompromised individuals have a higher risk of hospitalization and death from COVID-19 [5-8] due in part to their suboptimal responses to those vaccines [9-11].

Early in the pandemic, governments worldwide implemented a number of public health nonpharmacological interventions (NPIs) to ease hospital demand; slow down the spread of SARS-CoV-2; and protect high-risk groups, including immunocompromised individuals [12,13]. Interventions included *lockdowns,* travel restrictions, compulsory mask wearing, stay-at-home or other isolation policies, and physical distancing measures [12,13]. The US and UK governments advised immunocompromised individuals to maintain physical distancing, wear masks, avoid crowds, and keep up to date with COVID-19 vaccinations [14-16]. However, the population of immunocompromised individuals is heterogeneous—the degree of immunosuppression depends on the underlying disease or the duration of the condition and the type of immunosuppressive treatment [3,17-19]. Moreover, there is a lack of consensus on how severity should be categorized among immunocompromised individuals (eg, who should be considered moderately vs severely immunocompromised) [9-11,15]. To date, this heterogenous population, together with inconsistencies in how governments and physicians advised NPIs, has created uncertainty for immunocompromised individuals as to what they should be doing to keep safe [14,20,21]. While conducting interviews with the patient authors of this protocol (after protocol development), a common statement was that “life was never static—we (immunocompromised individuals) had to adapt to an ever-changing landscape.”

Although NPIs effectively reduce the risk of SARS-CoV-2 infection [22], the psychosocial impact of such measures is unclear [23]. While there were some cases in which immunocompromised individuals reported that NPIs had a positive impact on family life, health care access, and mental health [24,25], for many immunocompromised individuals, NPIs led to negative effects on mental and physical functioning in areas such as exercise, social support, independence, sleep, daily routines, anxiety, and continuity of care [23-29]. As an example of disrupted continuity of care, in the interviews conducted after protocol development, the patient authors noted that COVID-19–related restrictions caused delays in cancer diagnosis and subsequent treatment initiation. In addition, support from family members was disrupted when those family members were infected with SARS-CoV-2. The patient authors also described an increased sense of isolation caused by being unable to spend time with family members and friends. This view is reinforced in studies among the general population, which have also shown that NPIs were linked to negative impacts on work and career progression among participants from the United Kingdom, Italy, Malaysia, and Thailand [30].

Although government-imposed restrictions officially ended in 2022 for the United Kingdom and the United States (primarily due to effective vaccination programs in the general population), COVID-19–related hospitalizations and mortality remain disproportionately higher in immunocompromised individuals than in nonimmunocompromised individuals [31-33]. In the United Kingdom, people at high risk of severe COVID-19 outcomes (including immunocompromised individuals) were advised in September 2021 to take the same measures for avoiding SARS-CoV-2 infection as the general population, albeit with some additional precautions (no further updates have been issued as of November 2023) [15,34]. Nevertheless, current guidelines are challenging for many immunocompromised individuals, who perceive that their life cannot return to *normal* despite the rest of the world appearing to do so [35]. As of April 2022, UK data showed that 69% of individuals at high risk of severe COVID-19 were still taking extra precautions against COVID-19 and 13% were practicing physical distancing (including social isolation) and other self-protective behaviors [31].

Previous research on the psychosocial effects of physical distancing and social isolation for COVID-19 avoidance has focused on government-imposed restrictions, reflecting the *lockdown* period early in the pandemic [23,24,26-29]. There is little published research describing the broader impact of physical distancing and social isolation on the lives of immunocompromised adults, adolescents, and children (and their caregivers), whose distancing behaviors are no longer government imposed but rather voluntarily practiced based on medical recommendations or individual choices. In addition, the phenomenon of post–COVID-19 condition, also known as Long COVID, has not been extensively studied in immunocompromised individuals. While it has been established that post–COVID-19 condition has a detrimental impact on health-related quality of life (HRQoL) among the general population, the impact of post–COVID-19 condition among immunocompromised individuals is not well characterized [36,37].

### Objectives

Given these gaps in knowledge in the *postlockdown* setting, the EAGLE Study was designed to describe the association between physical distancing to avoid COVID-19 and HRQoL in immunocompromised adults, adolescents, and children (and their caregivers) in the United States and the United Kingdom.

In addition to these gaps in knowledge, most existing scales for measuring physical distancing do not target the extent to which a person may engage in various distancing behaviors specifically to avoid COVID-19 [38-44]. In 2020, Prachthauser et al [45] developed the Social Distance Scale (version 1), a brief, self-reported screening measure of adherence to social distancing and self-protective behaviors in pandemic situations; however, this scale was developed during the *lockdown* period and was aimed at the general population rather than high-risk groups. Furthermore, while the evidence for its measurement properties is limited, only its structural validity was assessed (solely via an item reduction–driven exploratory factor analysis, which was overly mechanical), and none of its resulting scales had good (*r*>0.9) consistency-based (Pearson correlation) test-retest reliability (2 scales had *r*<0.7); internal consistency, known-group validity, and convergent validity were not assessed. Moreover, the scale’s development and validation were only conducted with undergraduate students, with approximately 30% of the respondents excluded from analyses due to aberrant response patterns [45]. As such, it was deemed inappropriate for the context of the EAGLE Study, which aimed to rigorously assess the physical distancing behaviors of immunocompromised individuals of all ages at a point in time in which behaviors were practiced voluntarily rather than according to government mandates. Accordingly, the de novo Physical Distancing Scale for COVID-19 Avoidance (PDS-C19) was developed for this study to capture the extent of physical distancing behaviors. The psychometric properties of the PDS-C19 will also be assessed in the EAGLE Study. This paper summarizes the protocol of the EAGLE Study, entitled “An Observational Cross-sectional Survey to Describe the Association Between Socially Isolating to Avoid SARS-CoV-2 Infection and Health-Related Quality of Life in Immunocompromised Individuals and Nonimmunocompromised Individuals,” which was finalized and approved on October 17, 2022. The study sponsor is AstraZeneca, which contracted IQVIA to conduct the study (AstraZeneca study code: D8850R00013).

## Methods

### Patient Participation in Study Design

To inform how physical distancing behaviors and any associated burden should be captured from a patient’s perspective in future studies, 4 web-based qualitative focus groups [25] and 2 web-based asynchronous patient forums (personal communication by TM, 2023) were held with people at high risk of severe COVID-19, including immunocompromised individuals (and caregivers, where applicable).

The 4 web-based focus groups were held between April and July 2022. In these focus groups, people at high risk of severe COVID-19 were asked to describe the reasons and the extent to which they engaged in physical distancing and social isolation behaviors. Data from these focus groups were interpreted using deductive and inductive analysis [46]. Participants conveyed that these behaviors depended on their personal circumstances, the medical advice they had received, the availability of local services, and the prevalence of SARS-CoV-2 in their area. The impact on HRQoL of practicing these behaviors was also documented in these focus groups [25]. These insights and impacts were developed into a conceptual model of COVID-19 avoidance and protective behaviors that helped direct the development of the PDS-C19.

The 2 patient forums were held between July and August 2022 with people at high risk of severe COVID-19, some of whom had previously participated in the focus groups. These forums also helped direct the development of the PDS-C19 (personal communication by TM, 2023).

### Study Design

The EAGLE Study is a noninterventional, observational, cross-sectional survey of immunocompromised adults, adolescents, and children and the caregivers of immunocompromised children or adolescents living in the United States and the United Kingdom. Individuals who are not immunocompromised will be included as a reference group for nonformal comparisons and benchmarking purposes. The EAGLE Study is designed to capture the perspectives of individuals with and without immunocompromising conditions via self-report or child-caregiver (proxy) report at a point in time for each study participant during the COVID-19 pandemic. This will include some retrospective assessments to capture perspectives earlier in and before the COVID-19 pandemic. Physical distancing behaviors to avoid COVID-19 will be captured using the newly developed PDS-C19 instrument. HRQoL and health state utilities will be captured using previously validated instruments.

### Participants

In this study, immunocompromised individuals are defined as individuals with a moderate to severe immune compromise due to a medical condition or reported use of immunosuppressive treatments [16,47]. The categories of immunocompromising conditions and treatments are based on the UK government and US Centers for Disease Control and Prevention (CDC) guidelines [16,47]. Accordingly, based on their conditions, immunocompromised individuals will be grouped as follows: (1) blood cancers, (2) solid tumors (on active treatment), (3) solid organ or stem cell transplants, (4) end-stage kidney disease, (5) primary immunodeficiency disorders, (6) immunosuppressant treatments, (7) HIV infection (uncontrolled), (8) COVID-19 vaccine contraindications, and (9) other. While those with COVID-19 vaccination contraindications are not generally considered immunocompromised, they will be considered as immunocompromised for the purposes of the EAGLE Study because they are not protected by vaccination and, therefore, may remain susceptible to severe COVID-19 outcomes. The “other” category is included to ensure that those who do not know the exact categorization of their immunocompromising condition are not excluded from the study.

To be eligible to take part in the study, participants are required to meet all the inclusion criteria and none of the exclusion criteria (Table 1).

**Table 1:** Inclusion and exclusion criteria.

Criteria		Adults (aged ≥18 y)	Adolescents (aged 13-17 y) and children (aged 6 mo to 12 y)
**Inclusion criteria**			
	Individuals with immunocompromising conditions	At least 1 specified immunocompromising condition or treatment within 2 months before study enrollment	At least 1 specified immunocompromising condition or treatment within 2 months before study enrollment
	Individuals without immunocompromising conditions	No specified immunocompromising conditions, treatments, or history of an immunocompromising condition or treatment since January 2020	No specified immunocompromising conditions, treatments, or history of an immunocompromising condition or treatment since January 2020
	Caregivers of immunocompromised children or adolescents	Formal caregiver (ie, a parent or legal guardian) of the child included in the study Living (at least some of the time) with the child included in the study If the caregiver’s child is aged ≥5 years: willingness and ability to provide consent for their child to participate in the study in addition to assent from the child or adolescent	—^a^
	All individuals	Age of ≥18 years Residence in one of the eligible study countries Ability to read and understand English (United States and United Kingdom) or Spanish (United States) Willingness to complete a 30- to 45-min web-based survey related to feelings, health, living situation, and other descriptive questions within 1 week of the survey becoming available to them Willingness and ability to provide consent to participate in the study	Age of 6 months to <18 years Residence in one of the eligible study countries Have a formal caregiver who meets the caregiver eligibility criteria For children aged ≥5 years: Ability to understand English (United States and United Kingdom) or Spanish (United States) at a level typical for their age, with caregiver assistance if needed Willingness and ability to complete questions appropriate for their age about feelings and health and simple descriptive questions lasting <15 min, with caregiver assistance if needed Willingness and ability to provide assent to participate in the study
**Exclusion criteria**			
	All individuals	Participation in a clinical trial for experimental or investigational treatments for immunocompromising conditions and preventions or treatments for SARS-CoV-2 infection or COVID-19 Hospitalization or admittance to an inpatient facility at recruitment History of administration of tixagevimab or cilgavimab or any other passive immunization therapy for COVID-19 (history of vaccination against COVID-19 is acceptable)	Participation in a clinical trial for experimental or investigational treatments for immunocompromising conditions and preventions or treatments for SARS-CoV-2 infection or COVID-19 Hospitalization or admittance to an inpatient facility at recruitment History of administration of tixagevimab or cilgavimab or any other passive immunization therapy for COVID-19 (history of vaccination against COVID-19 is acceptable)

^a^Not applicable.

### Sample Size Calculation

Due to the descriptive nature of the study, no formal sample size or power calculations were conducted. Instead, an estimate based on an initial feasibility assessment was used to inform the sampling of key groups of interest and to achieve a reasonable representation of both the breadth of immunocompromising conditions and types and extent of physical distancing behaviors.

From an initial feasibility assessment conducted in early 2022 to test the survey approach for the EAGLE Study, 22% of adults with immunocompromising conditions reported that they were practicing physical distancing behaviors. Accordingly, a pragmatic sample size of 1400 immunocompromised adults was chosen for the EAGLE Study to ensure that the study would include approximately 300 socially isolating adult participants. Assuming that the prevalence of social isolation in adolescents and children is half that of the adult sample (11%), a sample size of 1818 adolescents and children was also chosen to ensure that approximately 200 adolescent and child participants would be currently socially isolating. The group of 1818 adolescent (aged ≥13 y) and child (aged <13 y) participants will be split in a ratio of approximately 1:1 to ensure roughly equal representation of adolescents and children. In addition to the immunocompromised participants, approximately 300 nonimmunocompromised adults, 100 nonimmunocompromised adolescents, and 100 nonimmunocompromised children (and the caregivers of these adolescents or children) are planned to be recruited to serve as an informal reference group for benchmarking purposes. Therefore, the total planned sample size for the EAGLE Study is 3718 (approximately 3218 immunocompromised and 500 nonimmunocompromised) participants, counting each adolescent- or child-caregiver pair as 1 participant. All adolescent and child participants will have a corresponding caregiver who, in addition to assisting or serving as proxy for their child, will complete some survey questions as a participant themselves. The planned sample size for each group is shown in Table 2.

**Table 2:** Planned sample size by age group and immunocompromised status^a^.

Age group (y)	Immunocompromised status, n			Total (N=3718), n
	Yes (n=3218)	No (n=500)		
Adults (≥18)	1400	300	1700	
Adolescents (13-17)	909	100	1009	
Children (0.5-12)	909	100	1009	

^a^All nonadults will have a corresponding caregiver (n=2018). The “children” age group comprises the following subgroups: older children (aged 8-12 y), young children (aged 5-7 y), toddlers (aged 2-4 y), and infants (aged 0.5-<2 y).

### Recruitment

**Figure 1:**
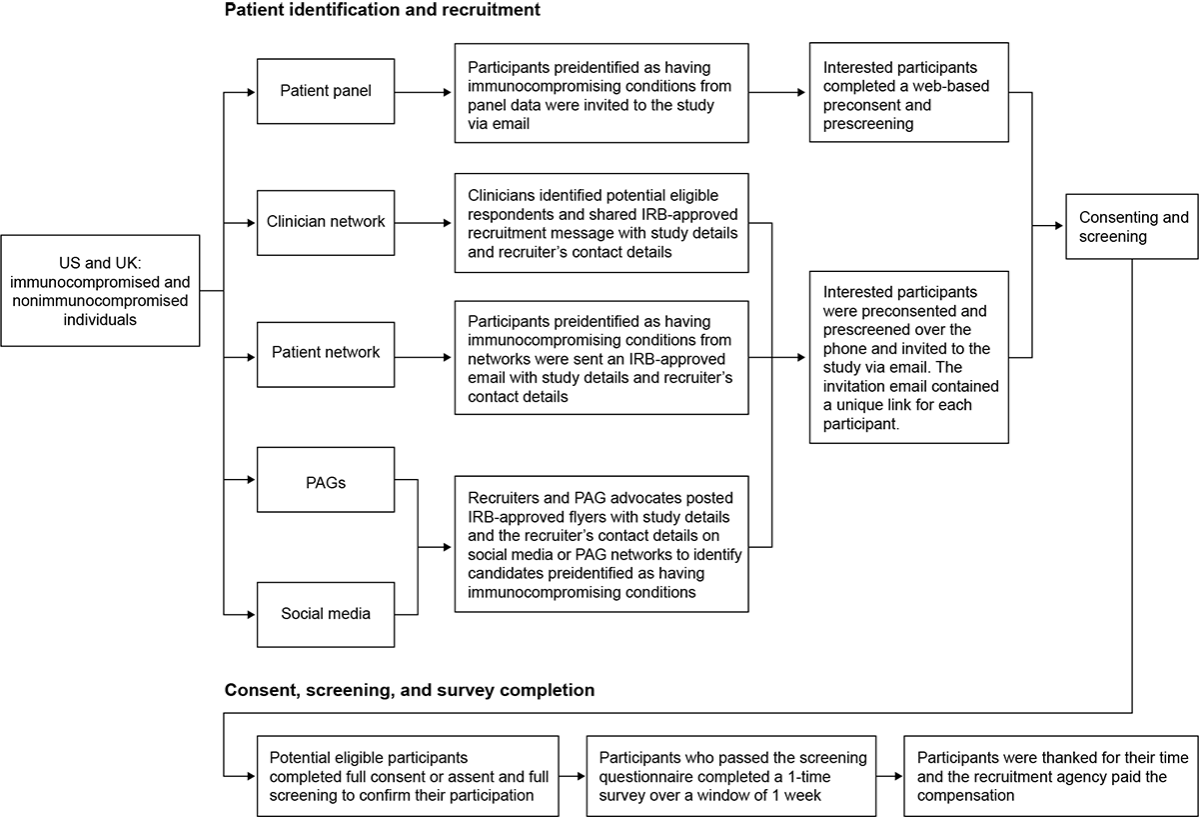
EAGLE Study flowchart. IRB: institutional review board; PAG: patient advocacy group.

Participants in the United States and United Kingdom are planned to be recruited via the patient recruitment agency Global Perspectives. To diversify participant recruitment, multiple direct-to-patient channels will be used (Figure 1). The eligible population will comprise 2 sets of participants: 1 set recruited through patient panels and networks (approximately 30%) and 1 set recruited through clinician referral, patient advocacy groups, and social media (approximately 70%). Child (or adolescent) and caregiver pairs will be recruited together (ie, both the child [or adolescent] and their caregiver must meet their respective eligibility criteria to participate in the study).

Potentially eligible individuals will be provided with information about the study and invited to participate via email with a unique link to a screening questionnaire to determine eligibility (Figure 1). Respondents who provide consent and pass the screening will be given access to the web-based study survey questionnaires. Completion is requested within 1 week to reduce the possibility of daily changes affecting survey responses while allowing sufficient time and pauses to complete the survey.

### Confirmation of Immunocompromising Diagnosis or Treatment

To verify that participants represent the spectrum of immunocompromising conditions that exist in real-world settings, a random subsample from the total study sample will be asked to provide confirmation of diagnosis (COD) via a medical document showing proof of either immunocompromising diagnosis or treatment. To calibrate the total number of COD invitations needed to achieve a 25% random sample from the immunocompromised population, the first 100 immunocompromised participants enrolled into the study will be invited to provide COD; the resulting acceptance rate will be used to calculate the number of COD invitations to extend. Participants who provide COD information will receive additional compensation at an institutional review board (IRB)–approved rate.

### Objectives and Outcomes

Broadly, the EAGLE Study has two parts: (1) psychometric validation of a new behavioral measure of physical distancing for COVID-19 avoidance (the PDS-C19) and (2) outcome analysis to explore the associations between the PDS-C19 measure and various HRQoL and utility measures collected as part of the survey.

Psychometric validation of the PDS-C19 will be conducted before outcome analyses using either an interim or randomly selected subset of approximately 1000 participants. The psychometric validation methods are detailed in the *Statistical Analysis* section.

The primary objective of the EAGLE Study is to describe the association between physical distancing to avoid COVID-19 and the HRQoL measures and health state utility values for immunocompromised adults. The secondary objectives are to describe the association between physical distancing and various HRQoL and utility measures among the other EAGLE Study participants. The specific study objectives and outcome measures are presented in Table 3.

**Table 3:** Outcome analysis: study objectives, population, and outcome assessments.

Objective and population				Outcome assessments
**Primary objective summary**				
	**To describe the associations between physical distancing to avoid COVID-19 and *HRQoL^a,b^*and *health state utilities***			
		Immunocompromised adults (aged ≥18 y)	The association between the PDS-C19^c^ score or scores and the following: SF-12v2^d^ [38], QDIS-7^e,f^, and DMOL^g^ item instruments [39] and the EQ-5D-5L [40] and SF-6D^h,i^ (based on the SF-12v2) instrument [41]	
**Secondary objectives**				
	**To describe the association between physical distancing to avoid COVID-19 and** ***HRQoL***			
		Immunocompromised adolescents (aged 13-17 y); older children (aged 8-12 y)	The association between the PDS-C19 score or scores and the PedsQL^j^ Generic Core Scales^k^ and DMOL item instruments	
		Young children (aged 5-7 y); toddlers (aged 2-4 y)^l^	The association between the PDS-C19 score or scores and the PedsQL Generic Core Scales^k^ [48]	
		Infants (aged 6 months-<2 y)^l^	The association between the PDS-C19 score or scores and the PedsQL Infant Scales [42]	
		Caregivers (aged ≥18 y)^m^	The association between the PDS-C19 score or scores and the PedsQL FIM^n^ instrument [49]	
	**To describe the association between physical distancing to avoid COVID-19 and** ***health state utilities***			
		Immunocompromised adolescents (aged 13-17 y)	The association between the PDS-C19 score or scores and the EQ-5D-5L instrument	
		Older children (aged 8-12 years); young children (age 5-7 y)^l^	The association between the PDS-C19 score or scores and the EQ-5D-Y^o^ instrument	
		Caregivers (aged ≥18 y)^m^	The association between the PDS-C19 score or scores and the EQ-5D-5L instrument	
	**To describe the association between physical distancing to avoid COVID-19 and** ***anxiety and depression***			
		Immunocompromised adults (aged ≥18 y) and adolescents (aged 13-17 y) and caregivers (aged ≥18 y)^m^ of immunocompromised adolescents and children	The association between the PDS-C19 score or scores and the HADS^p^ [43]	
	**To describe the association between physical distancing to avoid COVID-19 and work and school productivity impairment**			
		Immunocompromised adults (aged ≥18 y) and caregivers (aged ≥18 y) of immunocompromised adolescents^q^ and children	The association between the PDS-C19 score or scores and the WPAI+CIQ:SHP^r^ instrument [44]	
		Adolescents (aged 13-17 y)	The association between the PDS-C19 score or scores and the WPAI+CIQ:SHP (CIQ^s^ questions only) and PedsQL Generic Core Scales	
		Children (aged 5-12 y)	The association between the PDS-C19 score or scores and the PedsQL Generic Core Scales	
	**To describe the association between physical distancing to avoid COVID-19 and** ***HRQoL, health state utilities, anxiety and depression, and work and school productivity impairment***			
		Nonimmunocompromised adults, adolescents, and children and caregivers^m^ of immunocompromised adolescents and children	The association between the PDS-C19 score or scores and all outcomes as reported by the immunocompromised individuals	
**Exploratory objective summary**				
	**To describe the background frequency of symptoms similar to post–COVID-19 condition and describe the differences in background frequency of these symptoms between group or groups of immunocompromised individuals and group or groups of nonimmunocompromised individuals**			
		Immunocompromised and nonimmunocompromised adults, adolescents, and children	The frequency and differences in frequency of post–COVID-19 condition symptoms regularly over the past 4 weeks as measured using questions developed for this study Note that confirmation of post–COVID-19 condition diagnosis will not be captured in this study	

^a^Items in italics highlight key outcomes.

^b^HRQoL: health-related quality of life.

^c^PDS-C19: Physical Distancing Scale for COVID-19 Avoidance.

^d^SF-12v2: 12-item Short Form Health Survey version 2.

^e^QDIS-7: 7-item Quality of Life Disease Impact Scale.

^f^Mapi Research Trust [50].

^g^DMOL: Direct Measure of Loneliness.

^h^SF-6D: Short Form 6 Dimension.

^i^The SF-6D will be derived from the SF-12v2 instrument.

^j^PedsQL: Pediatric Quality of Life Inventory.

^k^Mapi Research Trust [51].

^l^Solely proxy reported; proxy version 1 for the EQ-5D-5L (caregiver’s opinion).

^m^For themselves, not via proxy.

^n^PedsQL-FIM: PedsQL Family Impact Module.

^o^EuroQol Group, the Netherlands [52].

^p^HADS: Hospital Anxiety and Depression Scale.

^q^Only school-related questions administered.

^r^WPAI+CIQ:SHP: Work Productivity and Activity Impairment Questionnaire plus Classroom Impairment Questions: Specific Health Problem (avoiding COVID-19).

^s^CIQ: Classroom Impairment Questions.

### PDS-C19 Measure

Targeted literature reviews conducted in April 2022 did not identify any existing fit-for-purpose scales for measuring physical distancing and social isolation behaviors specifically in relation to the avoidance of SARS-CoV-2 infection or COVID-19. Therefore, the de novo PDS-C19, a self- and observer-reported behavioral scale, was developed; the PDS-C19 will be used in the EAGLE Study to measure physical distancing to avoid SARS-CoV-2 infection and, thus, prevent COVID-19. The PDS-C19 contains questions that capture both the extent (frequency) of current physical distancing behaviors for a 4-week recall period and more distant retrospective recall of onset of current behaviors. These questions were based on distancing behaviors practiced by immunocompromised individuals to avoid SARS-CoV-2 infection. The behaviors were identified from public guidance for physical distancing, existing surveys from previous studies capturing data on social isolation during the COVID-19 pandemic, other social isolation scales, and the 4 patient focus groups [25]. The questions on physical distancing behaviors developed for the PDS-C19 were informed by and evaluated in 2 separate, web-based, debriefing patient forums involving 24 individuals at high risk of severe COVID-19 (23 patients in the first forum, of whom 22 patients returned in the second, follow-up forum [personal communication by TM, 2023]). Most participants in the second patient forum agreed that the physical distancing behaviors in the scale were relevant to their experience (n=19/22, 86.4%; personal communication by TM, 2023). The structural validity of the PDS-C19 will be assessed as part of this study. Further information about the PDS-C19 can be made available upon request to the corresponding author.

### Health Outcomes

Health outcome data related to the primary and secondary study objectives will be collected via previously validated age-specific HRQoL and related instruments (ie, the 12-item Short Form Health Survey version 2, Quality of Life Disease Impact Scale, Pediatric Quality of Life Inventory, Hospital Anxiety and Depression Scale, Direct Measure of Loneliness, and Work Productivity and Activity Impairment Questionnaire plus Classroom Impairment Questions: Specific Health Problem) and health state utility instruments (ie, the EQ-5D-5L, EQ-5D-Y, and Short Form 6 Dimension; Table 2). Patient-reported outcome (PRO) measures were selected by reviewing the social isolation literature to identify validated outcome measures used in similar studies and qualitative research describing the impacts of COVID-19 and social isolation. External clinical, health economic, and PRO experts (n=5) provided additional input. Direct input from immunocompromised individuals was also sought via the 4 patient focus groups. This helped conceptualize physical distancing and social isolation behaviors and their impact on HRQoL [25]. Regarding the PDS-C19 instrument, the PROs and the survey questions were evaluated in the 2 web-based, debriefing patient forums on HRQoL involving individuals at high risk of severe COVID-19. The aim was to assess the relevance and comprehensibility of the survey questions and implement feedback from forum members.

The PRO measures chosen for inclusion in the study cover a range of outcomes theorized to have a potential association with physical distancing behaviors. In addition, the chosen PRO measures are fit for purpose (ie, previously validated and appropriate for the context of use), well established, and available and licensable for use.

Finally, all the PRO measures to be used incorporate a recall period of ≤4 weeks, with most being 4 weeks and the remainder being 1 week or momentary (ie, current) at a time point within the 4-week PDS-C19 recall period.

### Exploratory Outcomes

A brief set of survey questions was developed to capture the frequency of signs and symptoms reported in individuals with symptoms similar to those of post–COVID-19 condition. However, to minimize bias in answering these questions, no survey question will directly ask about post–COVID-19 condition diagnosis, either self-reported or confirmed. At the time of protocol finalization in October 2022, there was no international consensus on the definition of post–COVID-19 condition; thus, these survey questions are based on post–COVID-19 condition guidance issued by the UK National Health Service, the US CDC, and a report from the US Department of Veterans Affairs [53]. As recommended by the National Health Service and US CDC, the questions related to symptoms similar to those of post–COVID-19 condition are written in lay language appropriate for self-reporting within a 4-week retrospective recall period.

### Study Survey Questionnaire

The study survey questionnaire is a 1-time, web-based, self- or proxy-administered set of questions comprising validated, age-appropriate PRO measures and study-specific questions that capture HRQoL outcomes, physical distancing behaviors, and key contextual data related to potential confounders (including demographic and socioeconomic factors). Input from the 4 web-based focus groups comprising individuals (including caregivers of immunocompromised children or adolescents) at high risk of severe COVID-19 informed the development of the survey questionnaire [25]. The languages of the survey questionnaire for study conduct are English (United States and United Kingdom) and US Spanish. The survey questionnaire is designed to be completed on any electronic device with an internet connection and is expected to take 30 to 45 minutes to complete.

PRO measures relating to the study’s primary objectives are placed toward the beginning of the survey questionnaire. Questions designed specifically for this study, which will collect more detailed and specific information, are positioned after (and within the same section as) the relevant PRO measures. Age-specific versions of the survey questionnaire for infants, toddlers, young children, older children, and adolescents have been designed with age adaptations to language and question content. Surveys are designed so that children aged ≥5 years can respond to questions on their own with assistance from their caregiver if needed, whereas all questions for toddlers (aged 2-4 y) and infants (aged <2 y) are directed toward caregivers only. The survey is structured so that children will complete questions about themselves first, allowing the caregiver to continue with other questions about the child or about themselves. Younger age groups have fewer questions to answer, whereas older age groups have progressively more questions to answer.

### Statistical Analysis

An interim or randomly selected subset of approximately 1000 participants stratified by immunocompromised status, age group, sex, and country will be partitioned from the main sample to serve as a psychometric validation sample for the PDS-C19. This subset will be further split into an exploratory factor analysis sample to identify underlying relationships between PDS-C19 items and a confirmatory factor analysis sample to test whether the scale structure or structures identified via the exploratory factor analysis is supported. After establishing the structural validity and scoring of the PDS-C19, the following psychometric properties will be evaluated: internal consistency, known-group validity (how well the scale can differentiate between groups that are known to differ), and convergent validity (how well the scale relates to other measures to which it is expected to be related).

**Figure 2:**
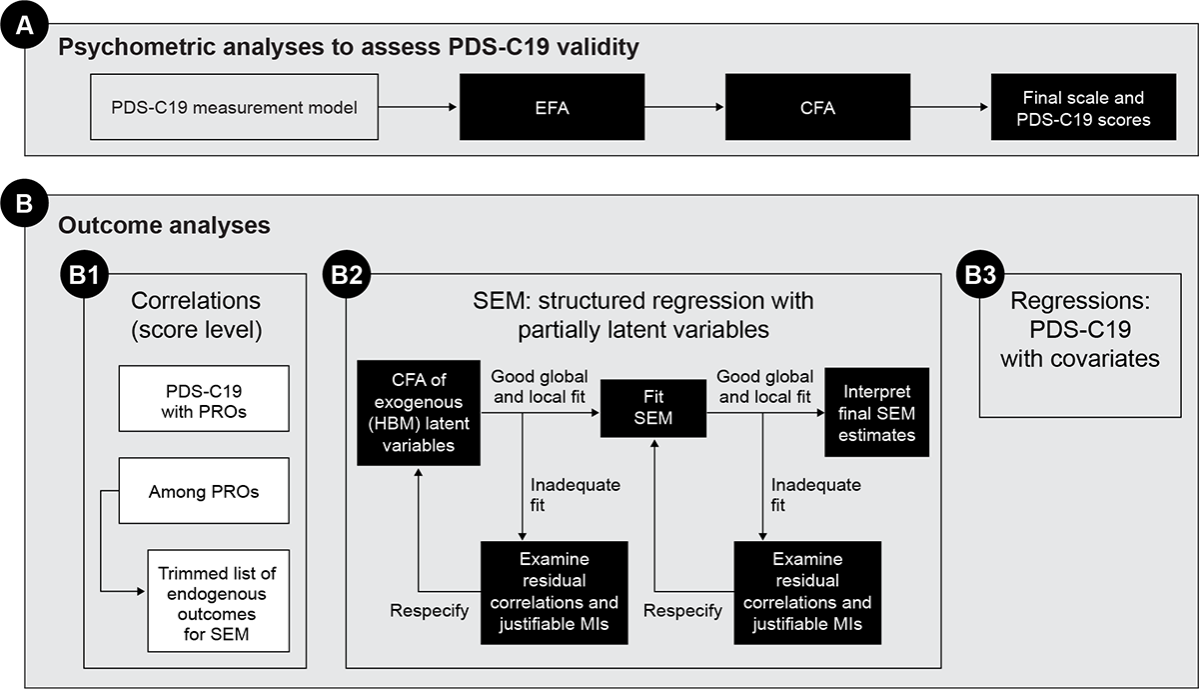
Outcome analysis flow diagram for primary and secondary objectives. CFA: confirmatory factor analysis; EFA: exploratory factor analysis; HBM: health belief model; MI: modification index; PDS-C19: Physical Distancing Scale for COVID-19 Avoidance; PRO: patient-reported outcome; SEM: structural equation modeling.

Figure 2 provides a flowchart for the overall statistical analysis using the final measurement model and scoring for the PDS-C19 developed as part of the psychometric analysis. After examining the Pearson correlations between PDS-C19 and the PRO scores (all scales are continuous), for immunocompromised respondents, prespecified structural equation models with partially latent variables will be fit to test hypothesized direct and indirect relationships between expected predictors of physical distancing (PDS-C19), other risk mitigation behaviors, and PRO measures. In addition, multiple regression analyses, with each PRO as a univariate dependent outcome, will examine the direct relationship between the PDS-C19 and the PROs controlling for other covariates (Figure 2).

Descriptive statistics for the various categorical and continuous health outcome scores and other survey items will be reported by age group and immunocompromised status. For each age group, descriptive analyses may also be reported univariately by immunocompromised category (immunocompromised participants only), country, and language (US and UK English vs US Spanish). The descriptive analyses may also be reported by categorized PDS-C19 scores. A statistical analysis plan for the EAGLE Study will be approved before conducting any analyses.

### Ethical Considerations

The EAGLE Study protocol and informed consent material were approved by the Western Institutional Review Board–Copernicus Group on November 15, 2022 (tracking 20226100), and those materials adhere to ethical principles consistent with the Declaration of Helsinki, guidelines for good pharmacoepidemiology practice, and applicable regulations and guidelines governing medical practice and ethics in the relevant countries. The UK regulatory authorities do not require local IRB approval for the conduct of this type of study. The final protocol and participant consent forms and all study recruitment and advertising materials will be implemented based on the IRB approval. In accordance with local regulations and ethical principles originating in the Declaration of Helsinki, participants will be required to provide informed consent electronically before being granted electronic access to the study and will be allowed sufficient time to consider participation. By signing and completing the electronic consent form, participants will consent to their data being used in the study unless they withdraw voluntarily for any reason and at any time. The consent form incorporated wording that complies with relevant data protection and privacy legislation. Consenting participants authorize the collection, use, and disclosure of their personal data by the third-party recruitment agency and by study team members, as necessary, for the purposes of the study. The consent form explains that study data will be stored in a secure computer database with confidentiality to be maintained in accordance with local data protection law or laws.

This study does not involve any safety objectives, and thus, adverse event reports will not be actively solicited. However, any incidental reports of adverse events regarding study sponsor products from study participants passively received by study staff during the course of the study will be reported to the study sponsor as per local country requirements and, as and when applicable, to the relevant regulatory authorities. After completing the survey questionnaires, participants will be compensated for their time based on a rate approved by the relevant IRB. Participants can discontinue at any time but will only be compensated after survey completion.

## Results

Participant recruitment and data collection were planned from December 2022 until June 2023, with all data analyses and reporting planned to be completed by December 2023. Results are planned to be submitted for publication in peer-reviewed journals in 2024-2025 and presented at national and international scientific conferences.

## Discussion

### Expected Findings

Following the end of government-imposed restrictions implemented to control COVID-19, the voluntary physical distancing behaviors practiced by immunocompromised individuals to prevent COVID-19 are still not well characterized. However, there is evidence suggesting that many of these individuals continue to practice physical distancing and social isolation behaviors similar to those required during government-imposed restrictions [25]. Emerging research published by the University of Liverpool during the EAGLE Study conduct has shown that immunocompromised individuals report higher levels of worry (due to COVID-19) and lower levels of mental health and well-being than the general population in the *postlockdown* setting [54]. The EAGLE Study will build on this research by comprehensively evaluating the associations between engaging in physical distancing behaviors for COVID-19 avoidance and HRQoL among immunocompromised individuals of all ages in the *postlockdown* era.

### Strengths

A key strength of the EAGLE Study is that its design was informed by input from immunocompromised individuals and from a group of individual expert advisors. Specifically, the self-reported physical distancing behaviors, HRQoL concepts, PRO instruments, and other survey items incorporated into the study were all informed by feedback from multiple stakeholders: clinical and health economic experts; specialists in HRQoL research; experts in the development, validation, and use of PRO instruments; and individuals at high risk of severe COVID-19 who contributed their insights over 4 focus groups and 2 forums. This study will provide the opportunity to use a randomly selected subsample of the EAGLE Study to refine the de novo PDS-C19 and its scoring via structural validity analyses and evaluate the instrument’s psychometric properties before its use in outcome analyses. The PDS-C19 will be a valuable tool for future studies and may serve as a framework that can be adapted for other infectious diseases.

Other strengths of the study design include the planned large sample size, inclusion of participants from 2 countries, inclusion of participants of nearly all ages, and the speed of data collection and subsequent analyses. A very large sample size will likely provide sufficient statistical power for the numerous analyses planned and should provide representation of key subgroups in this heterogeneous population. Due to this representation, we are more likely to obtain stable point estimates for these subgroups. In the midst of a pandemic, there is a need to balance credible study duration with the need for rapid research and data interpretation. The cross-sectional study design allows for more rapid data analysis and reporting than would be afforded by a longitudinal design while still providing valuable information about the relationship of interest.

Multiple channels of participant recruitment, a process that will involve prescreening and telephone calls with potential participants, and broad inclusion criteria are additional merits of the EAGLE Study. By requesting, collecting, and assessing confirmation of immunocompromising diagnosis information from a randomly selected large proportional subsample, it will also be possible to determine the level of representativeness of the study data regarding the broader immunocompromised population. In this study, HRQoL will be assessed using both validated, generic HRQoL instruments and instruments that are adaptable to specific conditions. Specific survey questions, all of which underwent multiple stages of development and testing, will be used to complement and help contextualize the main study findings. The study primarily focuses on the current (at the time of the survey: December 2022 to June 2023) and recent (within a 4-week period before the survey questionnaire completion) period for physical distancing and HRQoL outcomes. Choosing a recall period of within 4 weeks was considered appropriate to capture aspects of HRQoL and distancing behaviors less likely to be influenced by possible momentary fluctuations in health or daily activities.

### Limitations

In terms of evaluating the measurement properties of the PDS-C19 instrument, the cross-sectional study design precludes assessing the instrument’s test-retest reliability and sensitivity to change or what score change could serve as a threshold for meaningful change. For the EAGLE Study outcome analyses, inferences are also limited by the cross-sectional design. In the absence of longitudinal and other formal comparative data, causality or impact should not be inferred from any associations identified between physical distancing behaviors and other measures. At best, results can be deemed consistent with causal hypotheses. Nonetheless, the data from this study can serve as a strong basis for inferring possible causality and impact that could be demonstrated in future longitudinal studies.

As with all self-reported data, there is also the possibility of information bias, whereby participants may give inaccurate information based on their recall abilities, time frame of reference, and method of survey administration. Despite taking measures to ensure that the participant sample is representative of the real-world population, the influence of selection bias is possible such that the recruited participants might not be fully representative of the many categories and types of immunocompromising conditions (particularly rare ones) or of the nonimmunocompromised population. Furthermore, the web-based recruitment process may skew the sample toward individuals who are more familiar with electronic devices and browsing the internet. Another potential limitation is that the representativeness of subgroups may be reduced by the broad inclusion criteria (such as a wide age range and inclusion of many different immunocompromising conditions and categories). It is also possible that individuals with more severe immunocompromising conditions may be more invested in their underlying condition than those with milder immunocompromising conditions, meaning that those with a more severe immunocompromising condition are perhaps more likely to participate in research. In addition, we do not have prospectively collected baseline or other comparative data on physical distancing behaviors before the emergence of COVID-19, and there is no evidence of how physical distancing behaviors measured using the PDS-C19 are related to the risk of SARS-CoV-2 infection.

### Conclusions

This study will investigate the associations between physical distancing and social isolation to avoid COVID-19 and HRQoL and health utility measures in immunocompromised and nonimmunocompromised adults, adolescents, and children and the caregivers of immunocompromised adolescents and children. This study will also explore symptoms similar to those of post–COVID-19 condition in this population.

Following the finalization of this protocol, in May 2023, the World Health Organization declared COVID-19 an established and ongoing health issue that no longer constitutes a public health emergency of international concern [55]. However, there remains uncertainty around emerging and future SARS-CoV-2 variants, for which the transmission rates and case-to-fatality ratios are unknown or undercharacterized. In interviews with patient authors consulted for this protocol, it was noted that the end of government-imposed restrictions poses an even greater challenge for immunocompromised individuals, who must continue to take extra measures to protect themselves from infection. COVID-19 has substantially changed the way in which immunocompromised individuals live their lives.

This study will quantify and characterize the diverse HRQoL burden associated with physical distancing practices to avoid COVID-19 by immunocompromised and nonimmunocompromised individuals and caregivers of immunocompromised children and adolescents. It is anticipated that the data generated in the EAGLE Study will help inform future planning and recommendations by public health authorities and may be used directly in health technology assessments and health economic modeling. The data are also expected to be useful in developing guidelines for health care providers and immunocompromised individuals regarding the risks versus benefits of physical distancing to avoid COVID-19.

## Abbreviations

CDC: Centers for Disease Control and Prevention
COD: confirmation of diagnosis
HRQoL: health-related quality of life
IRB: institutional review board
NPI: nonpharmacological intervention
PDS-C19: Physical Distancing Scale for COVID-19 Avoidance
PRO: patient-reported outcome
